# A Mouse Model of Beta-Cell Dysfunction as Seen in Human Type 2 Diabetes

**DOI:** 10.1155/2018/6106051

**Published:** 2018-04-30

**Authors:** Jacqueline H. Parilla, Joshua R. Willard, Breanne M. Barrow, Sakeneh Zraika

**Affiliations:** ^1^Veterans Affairs Puget Sound Health Care System, Seattle, WA 98108, USA; ^2^Division of Metabolism, Endocrinology and Nutrition, Department of Medicine, University of Washington, Seattle, WA 98195, USA

## Abstract

Loss of first-phase insulin release is an early pathogenic feature of type 2 diabetes (T2D). Various mouse models exist to study T2D; however, few recapitulate the early *β*-cell defects seen in humans. We sought to develop a nongenetic mouse model of T2D that exhibits reduced first-phase insulin secretion without a significant deficit in pancreatic insulin content. C57BL/6J mice were fed 10% or 60% fat diet for three weeks, followed by three consecutive, once-daily intraperitoneal injections of the *β*-cell toxin streptozotocin (STZ; 30, 50, or 75 mg/kg) or vehicle. Four weeks after injections, the first-phase insulin response to glucose was reduced in mice when high-fat diet was combined with 30, 50, or 75 mg/kg STZ. This was accompanied by diminished second-phase insulin release and elevated fed glucose levels. Further, body weight gain, pancreatic insulin content, and *β*-cell area were decreased in high fat-fed mice treated with 50 and 75 mg/kg STZ, but not 30 mg/kg STZ. Low fat-fed mice were relatively resistant to STZ, with the exception of reduced pancreatic insulin content and *β*-cell area. Together, these data demonstrate that in high fat-fed mice, three once-daily injections of 30 mg/kg STZ produces a model of *β*-cell failure without insulin deficiency that may be useful in studies investigating the etiology and progression of human T2D.

## 1. Introduction

Type 2 diabetes (T2D) is characterized by hyperglycemia and is closely associated with obesity. Central to the development of T2D is failure of the pancreatic islet *β*-cell to adequately secrete insulin in order to maintain blood glucose levels within the normal physiological range [1]. This failure is contributed to by both impaired *β*-cell function and an absolute deficiency of *β*-cell mass. Insulin resistance is also important in the pathogenesis of T2D; however, frank diabetes develops only when *β*-cells fail to compensate for the increased secretory demand [2, 3].

A number of mouse models have been used to study T2D; however, not all recapitulate the broad spectrum of metabolic abnormalities observed in diabetic humans, as well as the numerous genetic and environmental influences contributing to the diseased state. For example, monogenetic models, such as db/db and ob/ob mice, do not mirror the polygenic aspect of human T2D, nor are genes encoding leptin or its receptor important contributors to T2D. Additionally, these models exhibit expansion of *β*-cell mass early in the course of disease, which does not parallel observations in humans [4]. Another widely used model is the high fat-fed C57BL/6 mouse, which exhibits numerous aspects of the diabetic phenotype typically seen in obese humans, including insulin resistance and hyperinsulinemia [5, 6]. However, diet-induced obese mice often do not demonstrate reduced glucose-mediated insulin secretion [7, 8], as is characteristic of human T2D, and, like db/db and ob/ob mice, exhibit expansion of *β*-cell mass.

Previous studies show that administration of streptozotocin (STZ) to high fat-fed mice results in decreased insulin concentrations [9, 10]. Both single (100 mg/kg) and multiple low (40 mg/kg) doses of STZ elicit such an effect on circulating (random) insulin levels, yet it is not clear whether the acute insulin response to glucose is also reduced. In one study, the insulin response to oral glucose was essentially absent in high fat-fed mice that received three consecutive injections of low-dose (50 mg/kg) STZ [11]. However, *β*-cell mass in these STZ-treated mice was reduced by 85%. Similarly, in another study, *β*-cell mass was halved following three consecutive injections of low-dose (40 mg/kg) STZ [9]. Thus, lower insulin levels following STZ treatment may be explained by reduced *β*-cell mass, rather than impaired *β*-cell function. While *β*-cell mass is indeed reduced in human T2D [4, 12–15], measurements of pancreatic insulin content demonstrate that insulin stores decline only as the disease progresses [16].

In this study, we sought to develop a nongenetic mouse model of the early defects in T2D, wherein glucose-mediated insulin secretion is reduced without significant loss of pancreatic insulin content. We used C57BL/6 mice, the gold standard background strain widely utilized in studies of diet-induced obesity and for generating genetically modified mouse models. We administered a 60% fat diet plus multiple low doses of STZ to mice then measured the insulin response to intravenous glucose to assess *β*-cell function followed by insulin in pancreatic extracts and tissue sections.

## 2. Materials and Methods

### 2.1. Animals

C57BL/6J male mice were from our colony at the VA Puget Sound Health Care System (VAPSHCS) in Seattle. At 5 weeks of age, mice were randomly assigned to receive diets containing either low (4% *w*/*w* or 10% calories from fat; no. D06041501P) or high (35% *w*/*w* or 60% calories from fat; number D12492) fat (Research Diets Inc.; New Brunswick, NJ). The fat composition of the low-fat diet was 23.5% saturated, 29.7% monounsaturated, and 46.8% polyunsaturated. The fat composition of the high-fat diet was 32.2% saturated, 35.9% monounsaturated, and 31.9% polyunsaturated. Mice had ad lib access to diets and water for the duration of the study. Body weight and fed plasma glucose levels were assessed weekly, with additional measures being made on days that included STZ injections. Studies were approved by the VAPSHCS Institutional Animal Care and Use Committee.

### 2.2. STZ Dosing Paradigm

After 3 weeks of low or high fat feeding, mice were randomized to receive either vehicle (citrate buffered saline, pH 4.5) or STZ (30, 50, or 75 mg/kg; Sigma-Aldrich, St. Louis, MO) once daily for three consecutive days by intraperitoneal injection. Thereafter, mice continued to receive low- or high-fat diets for 4 weeks.

### 2.3. Insulin and Glucose Tolerance Tests

Four weeks after vehicle or STZ injections, intraperitoneal insulin tolerance tests (1 IU/kg, ITTs) were performed in conscious mice fasted for 3.5 hours [8]. Tail vein blood was collected at 0, 15, 30, 45, and 60 minutes post insulin administration for glucose measurement. Two days later, intravenous glucose tolerance tests (1 g/kg; IVGTTs) were performed in pentobarbital (80 mg/kg) anesthetized mice fasted for 16 hours. Blood was collected prior to and 2, 5, 10, 20, 30, and 45 minutes post glucose bolus for glucose and insulin measurements [8]. The IVGTT procedure was chosen in order to specifically assess first-phase insulin responses. Pentobarbital anesthesia was utilized for the IVGTTs to minimize physiological stress associated with serial sampling to obtain sufficient plasma volumes for both insulin and glucose measures. While anesthesia can influence glucose and insulin dynamics, the effects of pentobarbital during a glucose tolerance test have been shown to be less pronounced than that of other anesthesia agents. Studies in rodents demonstrate that pentobarbital does not affect glucose levels, but can increase insulin levels [17–21].

### 2.4. Pancreatic Insulin Content

Following IVGTTs, mice were euthanized and a small portion of the pancreas was snap-frozen and homogenized in 0.18 M HCl/95% ethanol. The same pancreatic region from each mouse was extracted in order to avoid variability that may arise due to random sampling. The remaining portion of pancreas was used for histological assessments described below. Insulin content was measured and expressed as a proportion of total protein content.

### 2.5. Histological Assessments

Pancreas was excised, fixed in 10% neutral-buffered formalin overnight, paraffin-embedded, and sectioned at 4 *μ*m thickness. Deparaffinized sections were stained with anti-insulin antibody (1 : 2000; Sigma, St. Louis, MO) followed by Alexa Fluor 488-conjugated anti-mouse immunoglobulins to visualize islet *β*-cells, then counterstained with Hoechst 33258 (2 *μ*g/ml) to visualize cell nuclei. Morphometric analyses were performed using NIS-Elements AR 4.30.02 software on a Nikon E800 microscope system (Nikon USA, Melville, NY). The data collector was blinded to the diet and STZ status of each specimen. Histological assessments were made for all islets visible on each of two immunolabeled pancreas sections, averaging 32 ± 2 islets per mouse. To obtain the islet area, islets were identified morphologically and the edges were manually circumscribed using a multichannel image. Insulin- and Hoechst-positive areas were determined for each islet using pixel thresholding. The *β*-cell area was then calculated as Σ insulin area/Σ islet area × 100.

### 2.6. Glucose and Insulin Assays

Plasma glucose was determined using the glucose oxidase method. For ITTs, blood glucose was measured using an AlphaTRAK 2 glucometer (Abbott Laboratories, Abbott Park, IL). Insulin levels in plasma and pancreas extracts were determined using the Mouse Ultrasensitive Insulin ELISA (ALPCO, Salem, NH).

### 2.7. Calculations and Statistical Analyses

Data are presented as mean ± standard error of the mean (SEM) for the number of mice indicated. First-phase insulin responses during the IVGTT were computed as the ratio of incremental areas under the curve (iAUC) for insulin over glucose for 0–5 minutes. The surrogate index of insulin sensitivity, quantitative insulin sensitivity check index (QUICKI), was calculated from 16-hour fasting plasma glucose and insulin levels as follows: QUICKI = 1/[log(*I*_0_) + log(*G*_0_)], where *I*_0_ is fasting insulin (*μ*U/ml) and *G*_0_ is fasting glucose (mg/dl). Time-course data (body weight, weekly glucose, IVGTTs, and ITTs) were analyzed via a repeated-measures general linear model, while mean data were compared amongst study groups by analysis of variance. Post hoc analyses (LSD) were performed following both statistical tests. *p* ≤ 0.05 was considered statistically significant.

## 3. Results

### 3.1. Body Weight and Fed Glucose Levels

**Figure 1:**
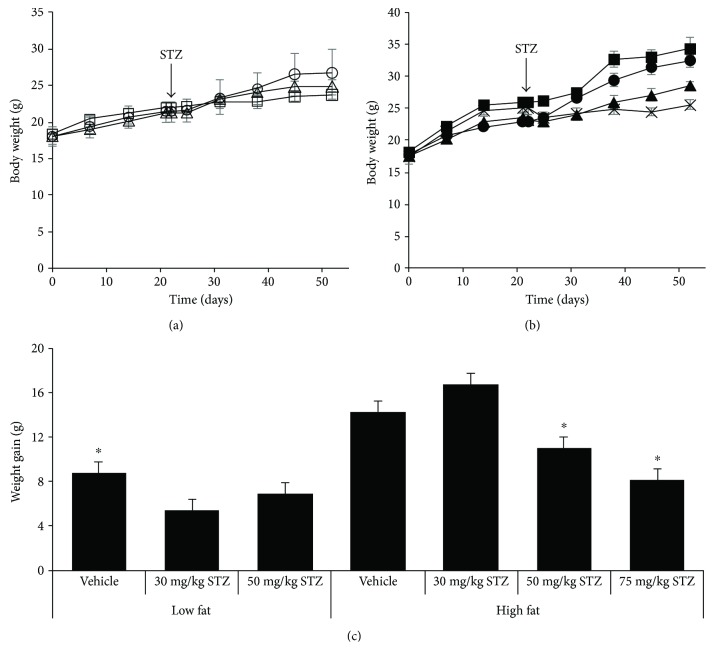
Body weight over time in low (a) and high (b) fat-fed mice treated with three consecutive once-daily doses of vehicle or 30, 50, or 75 mg/kg STZ. (c) Body weight gain from entry (day 0) to week 4 after vehicle or STZ treatments. Treatment groups are denoted as follows: low fat—vehicle, open circles (*n* = 4); low fat—30 mg/kg STZ, open squares (*n* = 4); low fat—50 mg/kg STZ, open triangles (*n* = 4); high fat—vehicle, closed circles (*n* = 4); high fat—30 mg/kg STZ, closed squares (*n* = 3); high fat—50 mg/kg STZ, closed triangles (*n* = 6); and high fat—75 mg/kg STZ, × (*n* = 3). Data are means ± SEM. ^∗^*p* < 0.05 versus high fat—vehicle.

At baseline, body weight did not differ amongst any of the groups of mice (Figures 1(a) and 1(b)). As expected, body weight during the course of the study was significantly greater in high- versus low-fat mice that did not receive STZ (*p* = 0.05). Within groups of low fat-fed mice, 30 mg/kg and 50 mg/kg STZ treatment did not alter body weight over time (Figure 1(a)). In contrast, low fat-fed mice that received 75 mg/kg STZ lost a significant amount of body weight and became severely hyperglycemic by the third week after STZ injections and had to be euthanized. Thus, data for this group of mice are not shown. In high fat-fed mice, 50 mg/kg STZ tended to lower body weight (*p* = 0.06 versus vehicle) and 75 mg/kg STZ significantly lowered body weight (*p* < 0.01 versus vehicle) by the end of the study period (Figure 1(b)). Thus, body weight gain over the duration of the study did not differ amongst low fat-fed mice, but was reduced in high fat-fed mice that received 50 and 75 mg/kg STZ compared to vehicle (Figure 1(c)). Weight gain in high fat-fed mice that received 30 mg/kg STZ did not differ from that in high fat-fed mice that received vehicle.

**Figure 2:**
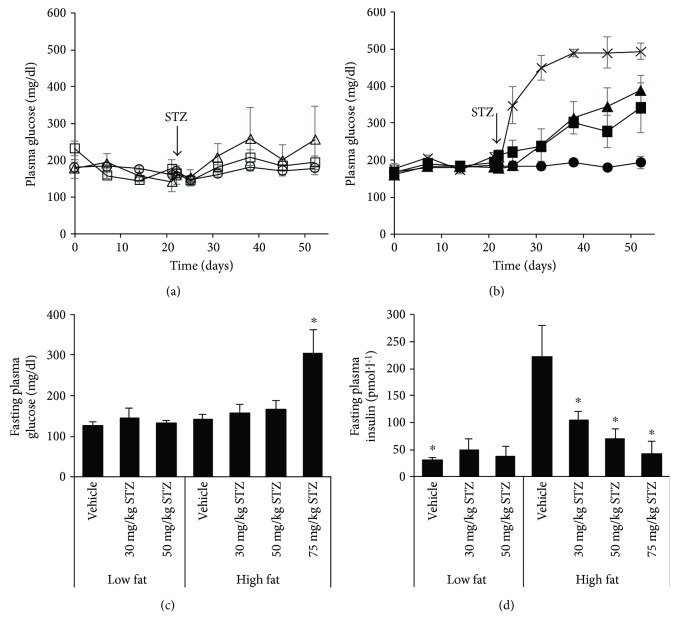
Fed plasma glucose over time in low (a) and high (b) fat-fed mice treated with three consecutive once-daily doses of vehicle or 30, 50, or 75 mg/kg STZ. 16-hour fasted plasma glucose (c) and insulin (d) levels four weeks after vehicle or STZ treatments. Treatment groups are denoted as follows: low fat—vehicle, open circles (*n* = 4); low fat—30 mg/kg STZ, open squares (*n* = 4); low fat—50 mg/kg STZ, open triangles (*n* = 4); high fat—vehicle, closed circles (*n* = 4); high fat—30 mg/kg STZ, closed squares (*n* = 3); high fat—50 mg/kg STZ, closed triangles (*n* = 6); and high fat—75 mg/kg STZ, × (*n* = 3). Data are means ± SEM. ^∗^*p* < 0.05 versus high fat—vehicle.

At baseline, fed glucose levels amongst groups of mice that went on to receive either low- (Figure 2(a)) or high-fat diet (Figure 2(b)) were not significantly different. Throughout the study period, fed glucose levels were comparable in low fat-fed mice receiving 30 mg/kg or 50 mg/kg STZ versus vehicle (Figure 2(a)). In contrast, high fat-fed mice displayed progressively elevated glucose levels with increasing doses of STZ (Figure 2(b); *p* = 0.07, *p* < 0.05, and *p* < 0.001 for 30, 50, and 75 mg/kg STZ versus vehicle, resp.).

### 3.2. Fasting Glucose and Insulin Levels and Insulin Sensitivity

Four weeks post-STZ or vehicle injections, fasting glucose (Figure 2(c)) and insulin (Figure 2(d)) levels were comparable amongst groups of mice fed low-fat diet. In high fat-fed mice, fasting glucose levels were similar four weeks post vehicle and 30 or 50 mg/kg STZ injections. However, high fat-fed mice that received 75 mg/kg STZ had significantly elevated fasting glucose levels, compared to mice from all other groups (Figure 2(c)). Further, fasting insulin levels were elevated in high versus low fat-fed mice that received vehicle (Figure 2(d)). However, in high fat-fed mice, all doses of STZ resulted in lower fasting insulin levels, compared to vehicle.

**Figure 3:**
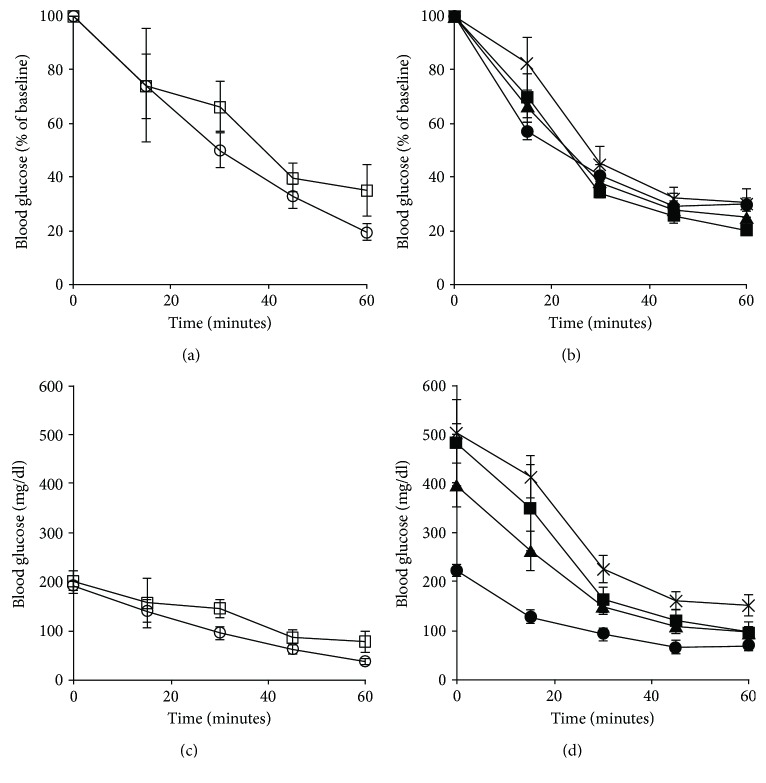
Blood glucose levels, as % of baseline and absolute values, during an ITT in low (a, c) and high (b, d) fat-fed mice treated with three consecutive once-daily doses of vehicle or 30, 50, or 75 mg/kg STZ. Treatment groups are denoted as follows: low fat—vehicle, open circles (*n* = 4); low fat—30 mg/kg STZ, open squares (*n* = 4); high fat—vehicle, closed circles (*n* = 4); high fat—30 mg/kg STZ, closed squares (*n* = 3); high fat—50 mg/kg STZ, closed triangles (*n* = 6); and high fat—75 mg/kg STZ, × (*n* = 3). Data are means ± SEM.

Insulin sensitivity, assessed by ITT four weeks post-STZ or vehicle injections, was comparable between low fat-fed mice that received vehicle versus 30 mg/kg STZ (Figures 3(a) and 3(c)). Of note, low fat-fed mice that received 50 mg/kg STZ became severely hypoglycemic during the ITT, necessitating early termination of the test, and thus ITT results were not obtained for this group. In high fat-fed mice, no differences in insulin sensitivity were observed amongst any of the groups when accounting for differences in baseline glucose levels (Figure 3(b)). Without adjusting for baseline glucose levels, all doses of STZ increased blood glucose during the ITT in high fat-fed mice (Figure 3(d); *p* < 0.05 versus vehicle). Similar results were obtained when insulin sensitivity was determined using the surrogate index, QUICKI (Table 1). In addition, QUICKI revealed a decrease in insulin sensitivity in high versus low fat-fed mice that received vehicle (*p* = 0.001). Since QUICKI is not derived from ITT data, it was possible to estimate insulin sensitivity in low fat-fed mice that received 50 mg/kg STZ. The latter data revealed no difference in insulin sensitivity when compared to low fat-fed mice that received vehicle.

### 3.3. Acute Insulin Response to Intravenous Glucose

**Figure 4:**
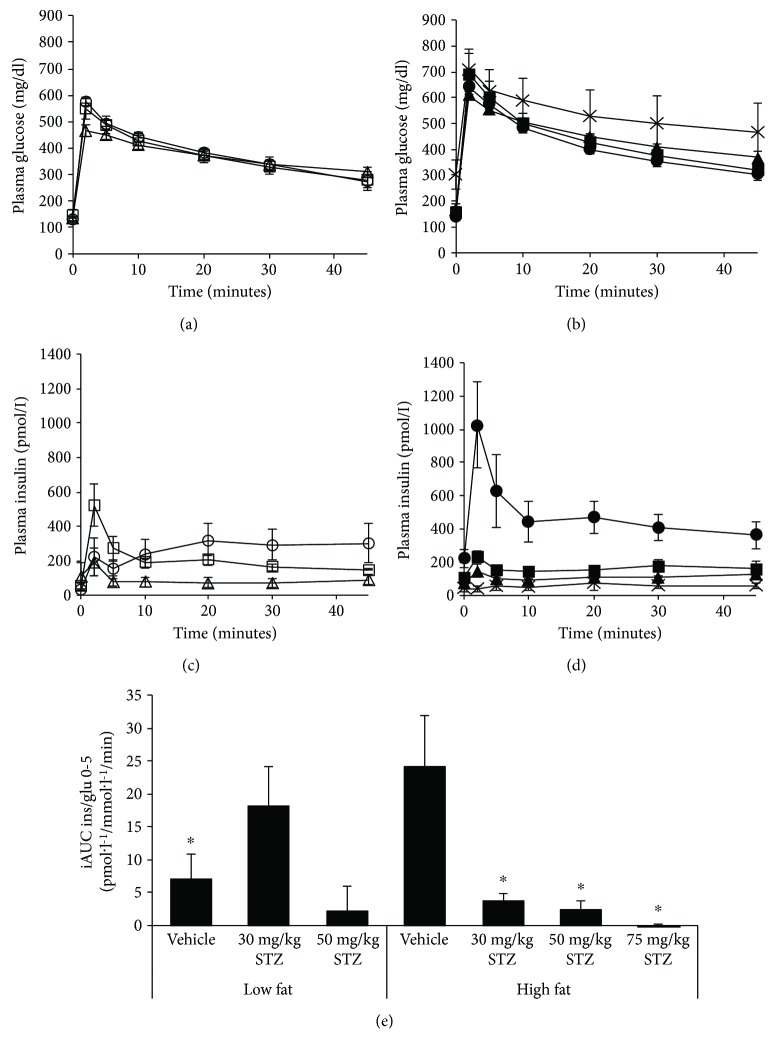
Plasma glucose (a, b) and insulin (c, d) levels during an IVGTT in low (a, c) and high (b, d) fat-fed mice treated with three consecutive once-daily doses of vehicle or 30, 50, or 75 mg/kg STZ. (e) First-phase insulin responses during the IVGTT, computed as the ratio of incremental areas under the curve for insulin/glucose from 0–5 minutes in mice treated with three consecutive once-daily doses of vehicle or 30, 50, or 75 mg/kg STZ. Treatment groups are denoted as follows: low fat—vehicle, open circles (*n* = 4); low fat—30 mg/kg STZ, open squares (*n* = 4); low fat—50 mg/kg STZ, open triangles (*n* = 3); high fat—vehicle, closed circles (*n* = 4); high fat—30 mg/kg STZ, closed squares (*n* = 3); high fat—50 mg/kg STZ, closed triangles (*n* = 6); and high fat—75 mg/kg STZ, × (*n* = 3). Data are means ± SEM. ^∗^*p* < 0.05 versus high fat—vehicle.

Four weeks post-STZ or vehicle injections, IVGTTs were performed to assess insulin secretion responses *in vivo*. Glucose levels during the IVGTT were similar amongst groups of mice fed low-fat diet (Figure 4(a)). In mice fed high-fat diet, glucose levels were similar in all groups except those that received 75 mg/kg STZ, which displayed significantly elevated IVGTT glucoses (Figure 4(b); *p* = 0.01 versus vehicle). Further, insulin levels throughout the IVGTT did not differ amongst groups of mice fed low-fat diet (Figure 4(c)). However, with a high-fat diet alone, insulin responses were elevated in mice that received vehicle, compared to all other groups (Figures 4(c) and 4(d); *p* ≤ 0.001). Finally, in high fat-fed mice, all doses of STZ resulted in diminished IVGTT insulin responses, compared to vehicle (Figure 4(d)).

Given that loss of first-phase insulin release is an early feature of T2D [22], we determined whether a high-fat diet plus low-dose STZ administration to mice could recapitulate this defect. First-phase insulin responses (iAUC) in low fat-fed mice tended to be higher following 30 mg/kg STZ (*p* = 0.08 versus vehicle), but not 50 mg/kg STZ (Figure 4(e)). High fat feeding resulted in significantly elevated first-phase insulin responses in mice treated with vehicle, compared to low fat feeding (*p* = 0.01 versus low-fat vehicle). Further, in high fat-fed mice, all doses of STZ significantly reduced first-phase insulin responses.

### 3.4. Pancreatic Insulin Content

**Figure 5:**
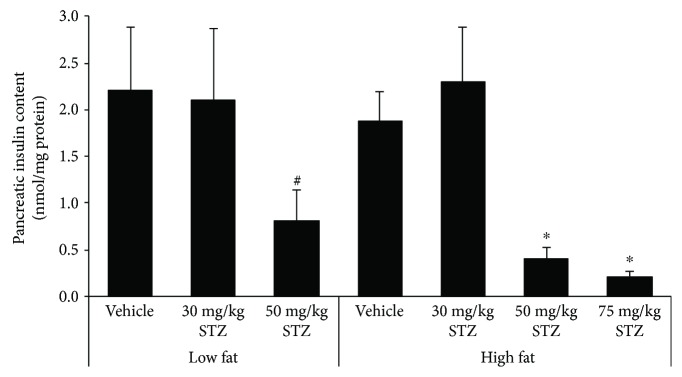
Pancreatic insulin content in low and high fat-fed mice treated with three consecutive once-daily doses of vehicle or 30, 50, or 75 mg/kg STZ. Data are means ± SEM. Low fat—vehicle, *n* = 4; low fat—30 mg/kg STZ, *n* = 4; low fat—50 mg/kg STZ, *n* = 4; high fat—vehicle, *n* = 4; high fat—30 mg/kg STZ, *n* = 3; high fat—50 mg/kg STZ, *n* = 6; high fat—75 mg/kg STZ, *n* = 3. ^#^*p* ≤ 0.05 versus low fat—vehicle, ^∗^*p* < 0.05 versus high fat—vehicle.

Pancreatic insulin content in low fat-fed mice was significantly reduced four weeks after 50 mg/kg STZ, but not 30 mg/kg STZ treatment, compared to vehicle treatment (Figure 5). Similarly, in high fat-fed mice, 50 mg/kg and 75 mg/kg STZ markedly decreased pancreatic insulin content, whereas 30 mg/kg did not (*p* = 0.35 versus vehicle), compared to vehicle treatment. Pancreatic insulin content did not differ between low and high fat-fed mice that received vehicle.

### 3.5. Pancreatic *β*-Cell Area

**Figure 6:**
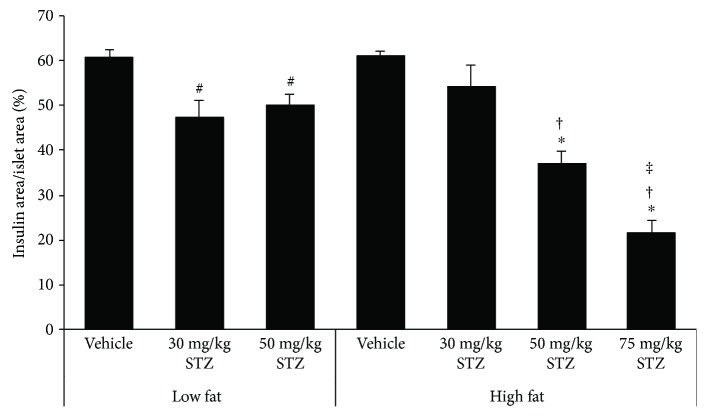
*β*-Cell area normalized to islet area, expressed as insulin positive area/islet area × 100%, in low and high fat-fed mice treated with three consecutive once-daily doses of vehicle or 30, 50, or 75 mg/kg STZ. Data are means ± SEM. Low fat—vehicle, *n* = 4; low fat—30 mg/kg STZ, *n* = 4; low fat—50 mg/kg STZ, *n* = 4; high fat—vehicle, *n* = 4; high fat—30 mg/kg STZ, *n* = 3; high fat—50 mg/kg STZ, *n* = 6; high fat—75 mg/kg STZ, *n* = 3. ^#^*p* < 0.05 versus low fat—vehicle, ^∗^*p* < 0.001 versus high fat—vehicle, ^†^*p* < 0.001 versus high fat—30 mg/kg STZ, ^‡^*p* < 0.01 versus high fat—50 mg/kg STZ.

The *β*-cell area in low fat-fed mice was significantly reduced four weeks after 30 mg/kg and 50 mg/kg STZ treatment, compared to vehicle treatment (Figure 6). Similarly, in high fat-fed mice, 50 mg/kg and 75 mg/kg STZ reduced the *β*-cell area, whereas 30 mg/kg did not (*p* = 0.14 versus vehicle), compared to vehicle treatment. The *β*-cell area did not differ between low and high fat-fed mice that received vehicle.

## 4. Discussion

In this study, we show that by administering a high-fat diet combined with multiple low doses of STZ to mice, we can reproduce the loss of first-phase insulin release that is a key early feature in the pathogenesis of human T2D [22]. Few nongenetic models that exhibit this specific aspect of human T2D currently exist, thus our *in vivo* mouse model described here could be useful for future work aimed at understanding the etiology and progression of *β*-cell dysfunction in T2D.

The critical role of *β*-cell dysfunction in the pathogenesis of T2D is well established. Following intravenous glucose injection in diabetic subjects, it has been demonstrated that the acute insulin response is markedly diminished [22, 23]. In our study, we saw a reduction in the first-phase insulin response when a high-fat diet was combined with multiple low-dose STZ injections. Additionally, the second-phase insulin response was also lower. This was accompanied by elevated fed glucose levels. Further, while 50 and 75 mg/kg STZ reduced pancreatic insulin content and *β*-cell area in high fat-fed mice, 30 mg/kg STZ did not, suggesting that the impairment of glucose-mediated insulin secretion with the lowest dose of STZ was due to a deficit in *β*-cell function, rather than *β*-cell mass. Indeed, *β*-/islet cell mass data from other studies parallel our findings with doses of STZ greater than 30 mg/kg in high fat-fed mice. Specifically, it was shown that *β*-cell mass in 3 × 50 mg/kg STZ-treated mice and islet mass in 3 × 40 mg/kg STZ-treated mice were reduced by 85% [11] and 50% [9], respectively. These studies also reported decreased insulin (glucose-stimulated and random circulating) concentrations following STZ treatment, which may be explained in part by reduced *β*-cell mass, rather than solely impaired *β*-cell function. The same holds true for our findings with 50 and 75 mg/kg STZ in high fat-fed mice. In human T2D, the deficit in *β*-cell mass has been found to be lower than 85%, ranging from 24% to 65% [4, 13, 15, 24–28]. In terms of pancreatic insulin content, the majority of human studies report a decrease in T2D subjects [15, 29–32]. However, when assessing insulin content within the first five years after diagnosis of diabetes, it was found that insulin content was unchanged in diabetic versus nondiabetic subjects; but, beyond five years, insulin content was reduced in diabetic subjects [16]. These data imply that insulin content in humans declines with disease progression. Thus, our mouse model where insulin content is not reduced in high fat-fed mice treated with 3 × 30 mg/kg STZ may be useful for studying *β*-cell defects that occur early in the disease process. It is important to acknowledge, however, that although insulin content and *β*-cell area were unchanged in our hands, we cannot rule out that *β*-cell mass may have been reduced in high fat-fed mice treated with 3 × 30 mg/kg STZ. Since our measures do not account for potential changes in pancreas weight with diet and STZ treatments, assessment of *β*-cell mass (i.e., *β* − cell area/pancreas′ area × pancreas′ weight) may yield results that are at odds with insulin content and *β*-cell area data. Of note, we generally saw a similar trend of 50 mg/kg and 75 mg/kg STZ to decrease both insulin content and *β*-cell area in low and high fat-fed mice, although the magnitude of the effect differed between the two measures. One reason for such discrepancies may be our pancreas sampling method, where the same pancreatic region from each mouse was extracted for all insulin content measures and another region was processed for histology.

While we observed a clear impairment of *β*-cell function in high fat-fed mice treated with STZ, insulin sensitivity in these mice was not reduced when assessed by the ITT, QUICKI, or fasting insulin level. This is somewhat unexpected given a previous report of decreased insulin sensitivity in high fat-fed mice that received at least three 40 mg/kg STZ injections [9]. Potential explanations for our discrepant data may be related to the use of different C57BL/6 substrains, as well as the age of mice and dose of insulin administered during the ITT. Gilbert et al. studied C57BL/6NCr mice, whereas we utilized C57BL/6J mice. Multiple genetic differences have been found between C57BL/6N and C57BL/6J substrains [33–36], some of which greatly influence the metabolic phenotype (e.g., nicotinamide nucleotide transhydrogenase [37–41]). While some responses to high fat feeding are similar amongst different mouse strains [42, 43], there exists heterogeneity amongst C57BL/6 substrains [8, 35, 44–46]; thus, it is possible that the metabolic response to STZ also differs. Such heterogeneity has been demonstrated in C57BL/6J versus ICR mice, where the latter were more susceptible to STZ-induced hyperglycemia [10]. With respect to age, mice were six months old at the beginning of the Gilbert et al. study, yet our mice were only 5 weeks old. It has been shown that in mice, both insulin sensitivity and glucose tolerance deteriorate with age [47–49]. Further, for the ITT, we utilized a dose of insulin that was greater than what Gilbert et al. used (i.e., 1.0 versus 0.75 IU/kg). Thus, perhaps if we had studied older mice and/or used a lower dose of insulin for the ITT, loss of insulin sensitivity may have been apparent. Moreover, a longer exposure to a high-fat diet may also increase susceptibility to insulin sensitivity in STZ-treated mice (as we have observed in our unpublished data). For the purposes of developing a model of impaired *β*-cell function, it may be desirable for insulin sensitivity to remain unchanged since it is a major regulator of the insulin response to glucose *in vivo* [50, 51] and thus can be a confounder for data interpretation. In low fat-fed mice, we found that 50 mg/kg but not 30 mg/kg STZ induced severe hypoglycemia following i.p. insulin administration. We speculate this may be due to defective counterregulatory responses, including an impaired glucagon response [52], although further studies are needed to better define the mechanisms responsible.

The effect of low-dose STZ on the metabolic phenotype differed in low versus high fat-fed mice. Interestingly, low fat-fed mice that received STZ did not exhibit reduced insulin secretion; in fact, 30 mg/kg STZ tended to increase the first-phase insulin response in low fat-fed mice. Moreover, fasting and fed glucose levels were unaltered in low fat-fed mice that received STZ, although insulin content and *β*-cell area were significantly reduced with 50 mg/kg STZ. The *β*-cell area was also reduced with 30 mg/kg STZ in low fat-fed mice. These data suggest that *β*-cells in low fat-fed mice treated with STZ do not face increased demand for insulin secretion and thus can afford a deficit in *β*-cell mass before any impairment in glycemic status ensues. Consistent with our data, it has previously been reported that low fat-fed mice are relatively resistant to the effects of low-dose STZ [9, 10], compared to high fat-fed mice. An exception to this is with use of 75 mg/kg STZ; that is, we found that in low fat-fed mice, 75 mg/kg STZ induced significant body weight loss and severe hyperglycemia by the third week after STZ injections. This suggests that there exists a critical threshold at which the majority of *β*-cells succumb to the toxic effects of STZ and can no longer control glucose levels. This threshold is higher in the setting of increased dietary fat, which might be explained by an initial expansion of *β*-cell mass; however, further studies are needed to elucidate this.

Our study design included mice that received 30, 50, or 75 mg/kg STZ. We found that three consecutive doses of 50 or 75 mg/kg STZ administered to high fat-fed mice induced a phenotype that was more akin to type 1 diabetes, where pancreatic insulin content was markedly diminished and thus glucose-mediated insulin secretion was almost absent. In contrast, 30 mg/kg STZ was sufficient to induce an impaired insulin response to glucose and hyperglycemia, without changing the body weight, insulin sensitivity, pancreatic insulin content, or *β*-cell area. Thus, in our hands, we feel that the combination of 30 mg/kg STZ and increased dietary fat in C57BL/6J mice creates an ideal model for the study of reduced first-phase insulin release in T2D. However, to be a reliable model in T2D research, the efficacy of 30 mg/kg STZ and 60% fat diet in inducing *β*-cell dysfunction must first be determined empirically in each laboratory, as even batch-to-batch variations in reagents and mice exist and can limit reproducibility. With this in mind, our model will be useful for evaluating the ability of drugs to improve insulin secretion because pancreatic insulin content is preserved. Previously, it was shown that when *β*-cell mass was dramatically reduced (by 85%) in STZ-treated high fat-fed mice, it was not possible to improve the insulin response to glucose with antidiabetic agents known to be insulinotropic in humans [11]. Finally, our model may also be attractive to researchers because it is relatively inexpensive and simple to employ, when compared to genetic models or those that involve a protracted period of diabetes induction.

## 5. Conclusions

By treating high fat-fed mice with three consecutive doses of 30 mg/kg STZ, we have developed a nongenetic model of impaired *β*-cell function, specifically with loss of first-phase insulin release as seen in the early stages of human T2D, as well as diminished second-phase insulin release which is a feature of more established T2D. Due to the potential for variability in metabolic responses to 30 mg/mg STZ and high-fat diet amongst different laboratories, it is imperative that the efficacy of both STZ and high-fat diet to induce *β*-cell dysfunction be evaluated prior to moving forward with studies. Overall, this mouse model should be useful for investigating approaches to prevent and treat T2D.

## Figures and Tables

**Table 1:** Insulin sensitivity, estimated using QUICKI, in low and high fat-fed mice treated with three consecutive once-daily doses of vehicle, or 30, 50, or 75 mg/kg STZ.

Diet	Treatment	*n*	QUICKI
Low fat	Vehicle	4	0.331 ± 0.010
	30 mg/kg STZ	4	0.318 ± 0.021
	50 mg/kg STZ	3	0.301 ± 0.030

High fat	Vehicle	4	0.256 ± 0.007^∗^
	30 mg/kg STZ	3	0.274 ± 0.009^∗^
	50 mg/kg STZ	6	0.292 ± 0.008^∗^
	75 mg/kg STZ	3	0.295 ± 0.016

QUICKI = 1/[log(*I*_0_) + log(*G*_0_)], where *I*_0_ is fasting insulin and *G*_0_ is fasting glucose. Data are means ± SEM. ^∗^*p* < 0.05 versus low-fat vehicle.
